# Comparison of Influenza Vaccine Effectiveness Estimates Using Test-Negative Prospective Enrollment and Electronic Health Record Data, United States, 2024–2025

**DOI:** 10.64898/2026.07.23.26358258

**Published:** 2026-07-27

**Authors:** Ashley M. Price, Callie McLean, Seana Cleary, Aleda M. Leis, Ivana A. Vaughn, Stacey L. House, Sam Ellsworth, Krissy Moehling Geffel, Louise H. Taylor, Manjusha Gaglani, Kempapura Murthy, Elie A. Saade, Christopher Ladikos, Vel Murugan, Joanna L. Kramer, Brian D. Williamson, Erika Kiniry, Emmanuel B. Walter, Natalie AB Bontrager, Sascha R. Ellington, Brendan Flannery, Jessie R. Chung, US Flu VE Network Investigators

## Abstract

**Background:**

Influenza vaccine effectiveness (VE) is assessed annually through prospective enrollment of patients presenting with acute respiratory symptoms in a test-negative study design. Influenza VE has also been estimated from electronic health record (EHR) databases by linking medical diagnoses, laboratory test results, and patient influenza vaccination. There are limited data on agreement between influenza VE estimates from prospective enrollment versus EHR databases.

**Methods:**

The US Influenza Vaccine Effectiveness Network prospectively enrolled outpatients meeting clinical screening criteria and collected respiratory specimens to determine influenza virus infection. Seven study sites also identified EHR databases that included diagnostic codes for outpatient encounters associated with medically attended acute respiratory illness (MAARI), clinical respiratory virus testing, and influenza vaccination status. Effectiveness of influenza vaccination against laboratory-confirmed influenza was estimated from both data sources using logistic regression models including patient age, study site, and month of illness as 100(%) x (1 – adjusted odds ratio), comparing influenza vaccination among laboratory-confirmed influenza-positive patients versus laboratory-confirmed influenza-negative patients.

**Results:**

From October 2024–April 2025, 2,016 (30%) of 6,793 prospectively enrolled patients and 75,885 (24%) of 282,444 EHR MAARI encounters had laboratory-confirmed influenza virus infection. Effectiveness of vaccination against laboratory-confirmed influenza was 36% (95% confidence interval [CI]: 26-44) among prospectively enrolled patients and 38% (95% CI: 36-39) among EHR MAARI encounters. Comparing influenza VE estimates from the two data sources, confidence intervals overlapped for all age groups except for adults aged ≥65 years: −3% (95% CI: −53–30) among prospective enrollment versus 35% (95% CI: 32–39) VE from EHR MAARI encounters.

**Conclusion:**

Overall, influenza VE estimates from retrospective EHR data were similar to VE estimates using the test-negative design with prospective enrollment. The age group-specific differences in estimated VE observed in US adults aged ≥65 years compared with younger age groups merit further investigation.

**Key Points:**

Retrospective EHR-based data and prospective enrollment of US patients using a test-negative design produced similar overall estimates of influenza VE during the 2024–2025 season.

## Full Text Availability

The license terms selected by the author(s) for this preprint version do not permit archiving in PMC. The full text is available from the preprint server.

